# Case report of ^18^F-FDG PET/CT features of hypoglycemic encephalopathy

**DOI:** 10.1097/MD.0000000000034025

**Published:** 2023-06-16

**Authors:** Xun-Ze Shen, Yan-Xing Zhang, Qiao-Ying You

**Affiliations:** a PET/CT Center, Shaoxing People’s Hospita, Shaoxing, Zhejiang Province, China; b Department of Neurology, Shaoxing People’s Hospital, Shaoxing, Zhejiang Province, China; c Department of Endocrinology, Shaoxing People’s Hospital, Shaoxing, Zhejiang Province, China.

**Keywords:** case report, diabetes, FDG PET/CT, hypoglycemia, hypoglycemic encephalopathy, magnetic resonance imaging

## Abstract

Hypoglycemia may cause diverse neurological manifestations, ranging from focal neurological deficits to irreversible coma. Severe and persistent hypoglycemia can lead to hypoglycemic encephalopathy (HE). Imaging findings of HE at different stages of ^18^F-FDG positron emission tomography/computed tomography (PET/CT) have rarely been reported. Herein, we describe a case of HE occurring in the medial frontal cortex, cerebellar cortex, and dentate nucleus using ^18^F-FDG PET/CT images from different periods. ^18^F-FDG PET/CT has a high value in displaying the lesion range and indicating the prognosis.

**Patient concerns:**

A 57-year-old male patient with type 2 diabetes (T2D) was transferred to the hospital with a history of unconsciousness for 1 night. The patient showed a significant decrease in blood glucose levels.

**Diagnoses:**

The patient was initially diagnosed with a hypoglycemic coma.

**Interventions:**

The patient subsequently underwent a comprehensive treatment. The ^18^F-FDG PET/CT examination on the fifth day after admission revealed a significant symmetrical fluorodeoxyglucose (FDG)-positive accumulation in the bilateral medial frontal gyrus, cerebellar cortex, and dentate nucleus. A follow-up PET/CT examination 6 months later revealed hypometabolism in the bilateral medial frontal gyrus and no abnormalities in FDG uptake in the bilateral cerebellar cortex and dentate nucleus.

**Outcomes:**

The patient condition was stable 6 months later, with a slow response, memory deterioration, occasional dizziness, and episodes of hypoglycemia.

**Lessons:**

HE lesions with a high metabolic status may be related to a metabolic compensation mechanism in response to gray matter loss. Some of the more severely damaged cells eventually die even after the blood sugar levels return to normal. Less damaged nerve cells can be recovered. ^18^F-FDG PET/CT has high value in indicating the lesion range and prognosis of HE.

## 1. Introduction

Glucose is the main energy source in brain tissue. Severe and persistent hypoglycemia may lead to long-term coma, seizures, and a myriad of other global and focal neurological deficits, resulting hypoglycemic encephalopathy (HE). The lesion area of HE is highly selective; it usually occurs in the cerebral cortex, hippocampus, cerebellum, basal ganglia, and other areas with high energy consumption.^[1]^ In the early stages, hypoglycemia triggers a series of protective responses to increase endogenous glucose production and reduce glucose disposal to restore normal glucose levels.^[2]^ ^18^F-FDG positron emission tomography/computed tomography (PET/CT) can detect changes in glucose uptake by nerve cells in patients with HE, and its dynamic changes help evaluate the patient condition.

## 2. Case presentation

A 57-year-old male patient with type 2 diabetes (T2D) was admitted to the hospital with a history of loss of appetite due to an unknown cause for several days and unconsciousness for 2 hours. Two hours prior, the patient was found unconscious by his wife during sleep at 4:30 am with purple lips and no limb voluntary movement. He was mentally and physically normal before going to bed at 9.30 pm on the previous day.

The patient had a history of T2D for 4 years and had been using a mixed protamine zinc recombinant human insulin injection 70/30 30 U subcutaneously (16 U in the morning and 14 U in the evening) combined with acarbose tablets (100 mg, 3 times a day) for a long time to reduce blood glucose levels without monitoring.

The patient was transferred to the emergency room of a local hospital. The blood glucose was measured as 1.4 mmol/L (normal range, 3.90–6.10 mmol/L; 1 mmol/L = 18 mg/dL), and the creatine kinase level was 210.9 U/L (normal range, 24–195 mmol/L). No obvious abnormalities were found in the blood sodium, troponin, prothrombin chart, or routine blood tests.

Head computed tomography (CT) examination showed no obvious abnormalities. The patient did not have an electroencephalogram.

At approximately 7 am, he was diagnosed with hypoglycemia and received symptomatic treatment such as promoting the revival of coma with Xingnaojing-injection (a Chinese patent medicine). The patient was not intubated and was transferred to our hospital. Upon admission, the patient was confused and delirious. The patient condition was not assessed using the Glasgow coma scale.

After admission, the patient blood glucose level rose rapidly, with a fasting blood glucose level of 6.14 mmol/L and 6.7% glycated hemoglobin A1c (normal range, 4.2%–6.2%), although he remained in an ongoing coma.

The patient blood glucose control regimen was changed to insulin aspartic injection (6 U in the morning, 4 U at noon, and 4 U in the evening) immediately before meals and insulin glycine injection (12 U before bed), combined with acarbose tablet 100 mg 3 times a day taken with the first bite of each meal.

**Figure 1.**
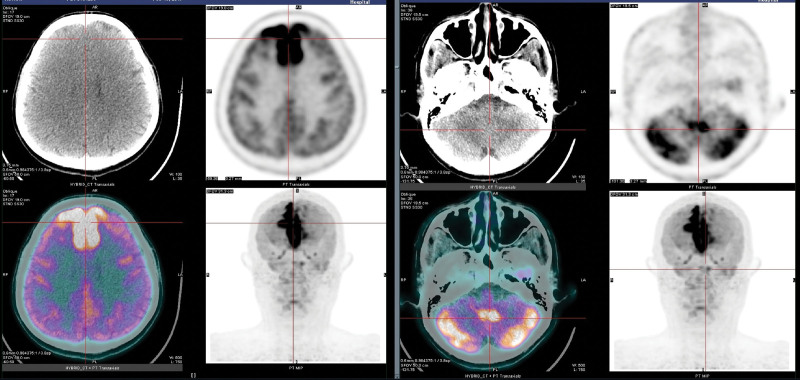
^18^F FDG PET/CT showed a symmetrical hypermetabolism in the bilateral medial frontal gyrus, cerebellar cortex, and dentate nucleus, especially in the bilateral frontal lobes. FDG = fluorodeoxyglucose, PET/CT = positron emission tomography/computed tomography.

Head MRI on the third day after admission showed high-signal lesions on diffusion-weighted images in the bilateral medial frontal gyrus and cerebellar cortex. On the fifth day after admission, ^18^F FDG PET/CT showed significant hypermetabolism in symmetry in the medial frontal gyrus on both sides, bilateral cerebellar cortex, and dentate nucleus, especially in the bilateral medial frontal gyrus with an maximum standardized uptake value of approximately 12.73 (Fig. 1). At the time of ^18^ F-FDG injection, the patient presented with fasting blood glucose levels corresponding to 10.1 mol/L. Clinically, the patient was awake, with the correct orientation of time and place and clear articulation, but was still unable to cooperate with bilateral deep and superficial sense examinations.

Based on these findings, HE was diagnosed.

After more than 10 days of preliminary treatment, the patient condition improved. However, he often showed irritability, accompanied by directional dysfunction (incapacity or misperception of the environment or one own condition) and an aggressive tendency, and was then transferred to a higher-level hospital for further treatment. The patient hypoglycemic regimen was modified again to “insulin glargine injection (8U/day), metformin tablets (0.5g, thrice/day), repaglinide tablets (1 mg, thrice/day), and acarbose tablets (100 mg, thrice/day),” which had been used ever since.

**Figure 2.**
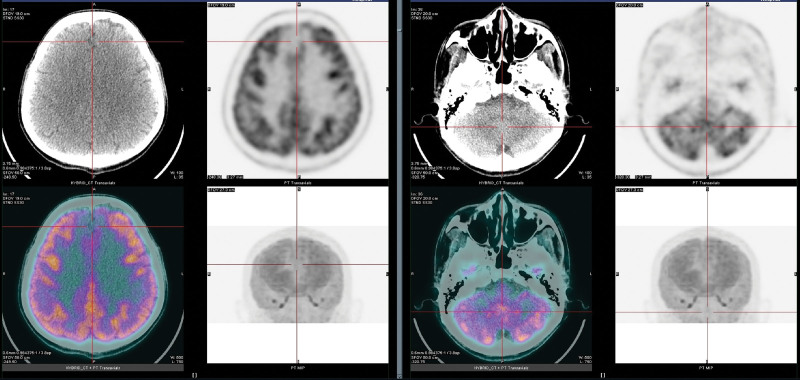
Half a yr later, the follow-up PET/CT showed hypometabolism in the bilateral medial frontal gyrus but no abnormal uptake of FDG in the bilateral cerebellar cortex and dentate nucleus. FDG = fluorodeoxyglucose, PET/CT = positron emission tomography/computed tomography.

The patient condition was stable 6 months later, with a slow response, memory deterioration, occasional dizziness, and episodes of hypoglycemia. The patient visited the hospital again for PET/CT review. Before the ^18^ F-FDG injections, the patient fasted for at least 6 hours. At the time of the radiopharmaceutical injection, the patient had blood glucose levels corresponding to 8.1mol/L. Follow-up PET/CT images showed hypometabolism in the bilateral medial frontal gyrus, but no obvious abnormalities in the uptake of fluorodeoxyglucose (FDG) in the cerebral cortex and dentate nucleus (Fig. 2 and Table 1).

**Table 1:** The timeline of the patient’s disease progression.

Timeline	The 1st d				The 3rd d	The 5th d	More than 10 d later	6 mo later
	4:30 am	6:30 am	6:30 am–7 am	8 am				
The event	The patient was found unconscious	The patient was taken to the emergency room of the local hospital	Blood glucose measurement and head CT examination	The patient was transferred to our hospital	Head MRI examination	PET/CT examination	The patient was transferred to a higher-level hospital for further treatment	PET/CT reexamination

PET/CT = positron emission tomography/computed tomography.

Ethical approval was not required as this was a case report of the patient clinical information. Written informed consent was obtained from the patient for publication of this case report and accompanying images.

## 3. Discussion

Hypoglycemia can occur at any age and is more common in elderly patients with T2D with a history of hypoglycemic drug administration or injection. The onset of hypoglycemia usually occurs at night; therefore, it is not easy to detect or recognize hypoglycemia in the early stages. If hypoglycemia occurs repeatedly, a plasma glucose concentration <2.8 mmol/L (50 mg/dL) for a long time (generally considered to be more than 6 hours) can cause serious or irreversible damage to the brain tissue. Even if the subsequent blood sugar concentration returns to normal, it can cause symmetrical damage to the bilateral basal ganglia, hippocampus, and cerebral cortex, leading to central paralysis, extrapyramidal hyperkinesia, mental decline, dementia, and other sequelae, known as HE. The lesion area of HE is highly selective. The present case involved the medial frontal cortex, cerebellar cortex, and dentate nucleus, which were closely related to the higher energy expenditure of the brain tissues in these areas. They are extremely sensitive to low blood glucose levels, and when blood sugar levels are low, these areas may be affected to varying degrees.^[1]^ HE is usually triggered by infection, diarrhea, or lack of food intake.^[3]^

The human brain consumes glucose as its primary energy source. Incipient hypoglycemia triggers a cascade of protective responses, including the suppression of endogenous insulin, stimulation of glucagon secretion, and sympatho-adrenal activation, thus increasing endogenous glucose production and reducing glucose disposal to restore normal glycemia levels. Hypoglycemic unawareness is associated with delayed onset and reduced magnitude of neuroendocrine responses to a falling blood glucose level.^[2]^ Studies have shown that the expression of the glucose transporters GLUT1 and GLUT3 increases in the brains of chronically hypoglycemic animals. Experiments on animal models of hypoglycemia have shown that increasing transporter density per unit surface area is not the only way to promote glucose uptake. Glucose uptake is also dependent on blood flow and the total surface area of the capillaries permitting transport.^[4]^ In terms of blood supply, delayed hypoperfusion occurs following moderate-to-severe hypoglycemia, although the initial physiological response to hypoglycemia is to increase cerebral blood flow to compensate for insufficient glucose.^[5]^

A comparative study conducted by Sampedro et al on ^18^F-FDG PET/CT images of 10 patients with impaired awareness of hypoglycemia (IAH) and 9 patients with normal awareness of hypoglycemia (all patients with type 1 diabetes) found that the FDG PET uptake of patients with T1D-IAH was higher than that of patients with T1D-normal awareness of hypoglycemia. Moreover, patients with IAH and T1D have abnormal hypermetabolism in brain regions strongly related to cognition.^[6]^

In the present case of HE, ^18^F-FDG PET/CT images on day 5 after admission showed significant hypermetabolism in the bilateral medial frontal gyrus, cerebellar cortex, and dentate nucleus, suggesting nerve cell damage in these areas. However, the follow-up PET/CT images 6 months later showed a significant decrease in FDG uptake in the lesions of the bilateral medial frontal gyrus, whereas no abnormality was observed in the lesions of the bilateral cerebellar cortex and dentate nucleus. This is the first report of HE with 18F-FDG PET/CT images in the early stages of the disease and at follow-up 6 months later. The following conclusions were drawn:

1. The lesions of patients with HE were not always deficient in FDG uptake after onset but had significantly increased glucose metabolism in the initial stage, which was also consistent with some literature reports.^[6,7]^
2. In the lesions, not all neurons damage is irreversible. In some areas with relatively minor damage, nerve cells can still return to normal, such as the bilateral cerebellar cortex and dentate nucleus.
3. Higher FDG uptake in the initial lesion area may indicate more severe damage and a greater likelihood of permanent damage, as in this case in the bilateral medial frontal lesion area.

The main pathological changes in HE are extensive degeneration and necrosis of neurons due to lack of energy, accompanied by extensive glial cell infiltration.^[1]^ The decrease in FDG uptake reflects reduced neuronal density.

## 4. Conclusion

HE lesions with a high metabolic status may be related to a metabolic compensation mechanism in response to gray matter loss.^[6]^ We believe that such compensation occurs not only in normal cells around damaged cells but also in damaged cells. Some of the more severely damaged cells eventually die even after the blood sugar levels return to normal. Less damage nerve cells can be recovered. The prognosis or neurological consequences of HE depend on the severity, duration of hypoglycemia, and response of the body to hypoglycemia.

## Authors contributions

**Conceptualization:** Xun-Ze Shen, Qiao-Ying You.

**Formal analysis:** Yan-Xing Zhang.

**Resources:** Xun-Ze Shen, Yan-Xing Zhang.

**Writing – original draft:** Xun-Ze Shen, Qiao-Ying You.

**Writing – review & editing:** Xun-Ze Shen.
